# Domestic Violence in Separated Couples in Italian Context: Communalities and Singularities of Women and Men Experiences

**DOI:** 10.3389/fpsyg.2018.01602

**Published:** 2018-09-03

**Authors:** Paola Cardinali, Laura Migliorini, Fiorenza Giribone, Fabiola Bizzi, Donatella Cavanna

**Affiliations:** ^1^Department of Educational Science, University of Genoa, Genoa, Italy; ^2^ASL3, Family Mediation Center, Genoa, Italy

**Keywords:** domestic violence, intimate partner violence, gender communalities, gender singularities, separated couples

## Abstract

Relationship breakdown and separation represent a critical aspect in domestic violence. Few studies have investigated domestic violence in separated couples. Moreover, there is a need for a more in depth analysis of gender differences that could enhance the comprehension of the phenomenon. The primary aim of this research was to analyze, through a qualitative approach, which kinds of domestic violence are characteristic or major in separated couples in the Italian context, where this phenomenon has not yet been sufficiently investigated. Participants are 60 separated couples (mean age: M = 48; F = 44) who attended a Family Mediation Center. A descriptive study was conducted using grounded theory methodology. A brief narrative task was administered to both ex-partners separately. The transcriptions were analyzed using NVivo 11 software. From data analysis, some themes emerged regarding typology of domestic violence specific of the separation context and shared by both men and women. The analyses of gender differences showed that there is a gender specific experience of domestic violence. Results highlight that women narrate both physical and psychological violence, while men relate only psychological abuse focused on limiting access to children. We discuss these findings in relation to possible appropriate gender specific intervention and prevention efforts.

## Introduction

Domestic violence represents an important concern for society; it is a widespread problem with adverse health consequences for all members of the family system. It has been defined as a range of actions that include physical and psychological aspects. Domestic violence against adults can be divided into three main types: psychological, physical, and sexual violence (World Health Organization [WHO], 2002). Inside the psychological abuse we find intimidation, constant depreciating and humiliating, and some controlling behaviors, such as isolating a person from their family and friends. Other forms of control are about monitoring a person’s movements and limiting their access to information or assistance. Physical aggression includes slapping, hitting, kicking, beating, and other violent behaviors. Sexual violence concerns forced intercourse and other forms of sexual coercion.

In the literature on domestic violence a lot of attention has been dedicated to different forms of intimate partner violence (IPV). IPV has been defined as a set of assaultive and coercive behaviors that includes threats, psychological abuse, physical aggression, and other hostile behaviors (Peisch et al., 2016). It occurs within an intimate relationship and shows consequences at physical, sexual, or psychological level and remains a prevalent global health problem (Catalano, 2000; Garcıa-Moreno et al., 2015).

Several studies have explored prevalence and determinants of IPV; according to Bucheli and Rossi (2017), attitudes toward men’s violence and women’s violence are correlated and can be due to the same factors. Pollack (2004) proposes a model about the intergenerational transmission of violence that is consistent with social learning theory (Bandura, 1969). The sociocultural perspective emphasizes the role of shared beliefs about gender roles and inequities in explaining differences in domestic violence between countries (Bell and Naugle, 2008).

Recent study shows that dehumanization reported by women represents a significant factor involved with partner abuse (Homa et al., 2017).

Intimate partner violence includes both verbal (e.g., insults, yelling, humiliation) and physical (e.g., pushing, shoving, choking) behaviors, that often tend to co-occur (Pepper and Sand, 2015). Psychological abuse comprises all devaluing or humiliating behaviors and forms of dominance and isolation (Cleak et al., 2018). Longobardi (2017), reporting the Centers for Disease Control and Prevention classification (Centers for Disease Control and Prevention, 2015), defines four main types of IPV: physical, sexual, stalking, and psychological. Physical violence has been defined as the intentional use of physical force to damage someone through behavior like scratching, pushing, shoving, or throwing. Sexual violence concerns sexual acts that are committed by another person without the consent of the victim. A special attention has been devoted to stalking, a “pattern of repeated, unwanted attention and contact that causes fear or concern for one’s own safety or the safety of someone else (e.g., repeated, unwanted phone calls, emails, or texts; leaving cards, letters, or flowers, etc.)” (Longobardi, 2017, p. 2039). Finally, psychological aggression includes the use of verbal and non-verbal communication to damage and/or to control another person (e.g., humiliation; limiting access to transport, money, relationships; threats of physical or sexual violence; and control of reproductive or sexual health). Johnson (2008) refined IPV types to reflect dyadic patterns within couples which views one partner’s use of violent and controlling behavior in combination with the other partner’s behavior.

According to some authors psychological IPV may be more mentally damaging than physical aggression (Crick and Grotpeter, 1995; Coker et al., 2002; Hellemans et al., 2015). Regarding this, Pepper and Sand (2015) found that only the perpetration and victimization of psychological violence were related with the overall feeling of oneself as a problematic person.

### Intimate Partner Violence and Relationship Breakdown

Relationship breakdown and separation represent a critical context for the study of domestic violence. The separation for a couple is a stressful life event and is associated with increased negative mental health and health problems. Therefore, the separation could be considered a risk factor for IPV (Logan and Walker, 2004). Furthermore in addition to the stressors, psychological problems may be experienced during a typical separation, especially women leaving abusive relationships often experience health and psychological problems related to the violence during the relationship. Separated women are more likely to experience violence than married women, and it is most common for women to experience violence from ex-partner. In the study of family relationship, IPV and parental separation are both considered major potential problems for children’s adjustment (Holtzworth-Munroe, 2011). It may be that violence follows separation, or the decision to separate is due to violence. International studies indicate that leaving a violent partner may increase the risk of more severe, or even fatal, violence. Indeed, the risk of violence increases during the process of separation when emotions are intensified (Cleak et al., 2018). In this process, destructive communication, such as throwing insults or bringing up events from the past, breeds strong relationship dissatisfaction. According to Johnston et al. (2005) study, the percentage of parents reporting domestic violence is higher among separating and divorcing parents than in the general population. In Beck et al. (2010) study, 85% of wives and 77% of husbands reported abuse (including emotional abuse and coercive control) during separation. Literature reveals that male partner violence or abuse is a statistically significant predictor of the female partner’s decision to separate (Hardesty, 2002). IPV is one of the main reasons given by couples seeking divorce (Amato and Previti, 2003; Gravningen et al., 2017).

It is well established that homicide rates are higher for women who have separated from their partners than for women in ongoing relationships (Hotton, 2001), this heightened risk of homicide following a separation is not found for men (Johnson and Hotton, 2003). However, according to DeKeseredy et al. (2004), separation may prevent or reduce the likelihood of physical assaults and emotional abuse against some women by their former partners. Separation may protect women from control-motivated assaults or from emotional abuse (Babcock et al., 2004).

### Gender Differences in IPV

As already highlighted in separation context, some studies underline some differences in IPV according to gender. In the specific context of relationship breakdown, men could see women’s decision about separation as a challenge, which makes them turn to violence as a mechanism to reestablish the culturally prescribed gender domination (Flake and Forste, 2006). According to Straus (2006), the most persistent and controlling forms of violence are perpetrated by men, this seems to confirm that IPV patterns could differ by gender. Also recent studies argue that women are not as violent as men and are more likely to use resistive or defensive violence (Holtzworth-Munroe et al., 2010; Carney and Barner, 2012). According to Caldwell et al. (2012), gender has a significant role in IPV because it is highly correlated with power. However, past findings have pointed that men and women tend to have different patterns of reporting of IPV; in particular men tend to under-report their own IPV perpetration while women are more likely to under-report their IPV victimization (Ko Ling, 2011). Men were more likely than women to be reported as using violent behavior like pushing, clutching, shoving, dragging, and choking, all fairly serious violent actions (Melton and Belknap, 2003; Ross and Babcock, 2015). In the specific context of separation, literature underlines a significant gender difference in the proportion of men and women citing domestic violence as a reason for the breakdown of their relationship (Gravningen et al., 2017).

Some studies highlight relational nature of IPV: men seem to be engaged in violence perpetration against non-violent partners at higher rates than women. Women more frequently perpetrated violence and control behavior in relationships with violent and/or controlling men (Coker et al., 2000; Mennicke and Kulkarni, 2016). Some studies underline that one area that has yet to be sufficiently explored is whether men and women agree on the acts, behaviors, and attitudes that comprise IPV in general (O’Campo et al., 2017), this is even more significant in case of separation. For these reasons, in the present study, we aim to fill the gap in the literature about similarities and differences in women’s and men’s experience of domestic violence during the separation process.

### Domestic Violence and Italian Context

In Italy, domestic violence is a widespread phenomenon. Domestic violence in Italy is a social reality at odds with the national ideology of family unity and cohesion. Perhaps this contradiction accounts for the scarcity of Italian research about IPV (McCloskey et al., 2002).

The National Institute of Statistics conducted a study in 2014 about domestic violence that provides some clues to its prevalence: 6.788 women suffered some form of physical or sexual violence during their lives. 20.2% of these women suffered physical violence, 21% sexual violence, 5.4% more severe forms of sexual violence such as rape and attempted rape. 13.6% suffered physical or sexual violence from partners or former partners (2.8 million), 5.2% (855,000) from current partners and 18.9% (2.44 million) from former partners. Most of the women who had a violent partner in the past left him because of the violence (68.6%). 41.7% of cases this was the main reason for relationship breakdown, for 26.8% domestic violence was an important element in the decision.

Separated or divorced women endured more physical or sexual violence than others (51.4% against 31.5%) (National Institute of Statistics, 2014). However, no data are available about men.

Experts have brought attention to the complexity and specificity of domestic violence associated with divorce. This topic needs to be investigated with particular attention to be contextualized with the mediation practices. Handling mediation cases with a history of domestic violence is one of the most controversial issues in the field of divorce mediation (Ballard et al., 2011; Pokman et al., 2014). However, it is an important topic because a significant number of separated couples, engaged in mediation intervention, report IPV and abuse (Rossi et al., 2015). Currently, there is a great deal of variation in how cases with IPV are handled by mediators. Some programs exclude violent cases from mediation, others simply conduct mediation as usual (Holtzworth-Munroe, 2011).

While in United States, associations provide significant guidance about case of domestic abuse that appears in mediation intervention, in Italy there are no explicit guidelines. However, one of the first laws that introduced the intervention of mediation is the Law number 66 of 1996, which reformed sexual violence and also took into account domestic violence; it suggests the intervention of a family mediator to protect the family relationship. In 2001, the Law number 154 about measures against violence in family relations introduces the express possibility for the judge to suggest mediation to hostile partners. With the Law number 54 of 2006, family mediation has been formally recognized as one of the tools that the judge can indicate in the treatment of cases of separation. This law provides that the judge, with the consent of the parties, can postpone the adoption of measures to allow spouses, using experts, to undertake a process of mediation to reach an agreement, with particular reference to the protection of moral and material interest of the children.

The analysis of family relationships in the Italian context should be made, taking into consideration the transformations of recent decades. The popular portrait of Italy as a country in which “family matters,” and the insistence of personalities with high public visibility on the importance of family integrity, are not matched by separation and breakdown rates. The transformations occurring in family relationships, on a psychological and social level, indicate a widespread and pervasive “fragility” of relations and their meaning. With regard to marital instability, National Institute of Statistic’s study about separation conducted in 2015 in Italy underlines that there was a substantial increase in the number of divorces that reached 82,469 cases (+57% compared to 2014). It is important to remember that in 2015, for the first time in Italy, two important regulatory changes concerning the dissolution of conjugal unions (law no. 132/2014 and law no. 55/2015) became operational. Much more moderated, and in line with the trends in previous years, is the increase in separations (91.706, +2.7%) compared to 2014. At the time of separation, husbands are an average 48 years old and wives 45 years old (National Institute of Statistics, 2015). However, the fragility of family living appears to constitute an existential condition, strongly connected with the uncertainty of modern society. In most of the Western world, a small number of people characterizes the nucleus of many families, especially in the urban context. This may constitute a risk factor for family isolation implying reduction of cultural, relational, and economic resources (Canvin et al., 2009). Moreover, families are particularly vulnerable to transitions and changes, particularly with respect to instability and precariousness of relationships. Furthermore, gender relations represent a modern challenge for the family, which remains primarily organized according to cultural determinants that define gender characteristics and differences. Some studies underline the matrifocal element of the Italian family unit. Women are perceived as devoted to family tasks and manage the housework, and men earn the income (Evertsson and Nermo, 2004). Rania et al. (2015) propose an image of paternity in Italian context slowly changing and redefining: new fathers seem to be more involved in the care of the children but mainly in recreational and executive activities, whereas mothers have a more active and organizational role than fathers. The family dynamic, particularly with minors, benefits a more stable structure (Migliorini et al., 2011, 2015) and continuity of relationships, even within the current dynamic of family setups (Garfinkel et al., 2001).

### Aim of the Current Study

No studies were found that reported IPV analyses among separated couples in Italy. The present work aims to increase the knowledge on:

– what types of experienced IPV are characteristic of separated context,
– what kind of experienced domestic violence are common or gender-specific in men’s and women’s narratives.

## Materials and Methods

### Participants

Participants are 60 separated couples. The average age of men is 48 years old and 44 years for women. The majority of the participants are graduates (55% of male; 43% of female) and employed (40% of men are workmen, 30% of women are office workers). Before breakdown, 70% had been married, while 30% cohabited. The average duration of the union was 12 years. All couples have one or more children.

### Materials

Because of the lack of existing research on this topic, we chose a qualitative study design. In recent years, psychosocial researchers have become increasingly aware of the need to improve qualitative methods in studies to understand the phenomena from the point of view of those who experience the situation. In addition to collecting demographic data (age, gender, educational level, current employment status), during their first meeting with the operator of Family Mediation Center, participants were asked to complete a narrative task. In accordance with the methodology already used in previous research (Tani et al., 2016), participants were requested to think about the history of their relationship, and briefly describe the main characteristics of the relationship with their ex-partner. Researcher through specific questions introduced the narrative: “Could you speak about the relationship with [partner’s name] in your own words and without my interrupting you with any questions or comments? What kind of person [partner’s name] is? How are you getting along together?”

The task is a stimulus that can facilitate the reflexive function. The participants had to exercise his/her awareness on themselves, on their ex-partner, and on the relationship between them. They also have to operate an integration between the emotional level and the cognitive level; between sensations emerging during the story and memories.

### Procedure

The project has been presented to the couples that began the mediation intervention. Participants were asked to fill out a brief socio-anagraphic schedule and informed consent. We include only couples in which both partners agree to participate. All narratives were audio taped during the first meeting and verbatim transcript. We chose to collect data in a Family Mediation Center in a medium-sized city in the northwest of Italy. The Center provides mediation services to divorcing or separating parties who have been court- or self-referred. All participants took part on a voluntary basis.

### Data Analysis

A grounded theory approach (Glaser and Strauss, 1967) was selected for the present study. We use as prevalent the objectivist approach because of the descriptive and explorative nature of the aims. The transcripts were analyzed with an iterative process of collecting and examining data (Charmaz, 2005). Data were compared from common teams using NVivo11 software. The narrative transcripts were coded privately and independently by two researchers using a codebook, and coding scheme for emerging themes or recurring domains of meanings across the narratives (Lofland and Lofland, 1995; Rossman and Rallis, 1998). All disagreements’ were discussed, and a code was agreed on. The software was used to organize the coded statements into nodes containing similar concepts and hierarchies of categories and subcategories. The data analysis generated some graphical representations about the main topics. The quotes inserted in the results were chosen from narratives to best represent the core emerging themes. The quotations were checked carefully to ensure that the meanings were preserved in the form that they were presented by the participants.

## Results

The analysis of the narratives in separated couples underlines some forms of domestic violence. We organized these materials in three main aspects: (1) the domestic violence experienced by both partners, (2) the domestic violence experienced exclusively by women, and (3) the domestic violence experienced exclusively by men.

As regards domestic violence, narratives highlight some characteristics common to the two groups. Both men and women reported domestic violence related to psychological abuse. In particular narratives analysis brings out seven sub-categories: limiting access to friends, oppression, verbal abuse, yelling, threats, slandering, and humiliating, that are briefly described in **Table 1**.

**Table 1:** Categories of domestic violence present in both women and men narratives.

Categories	Description
Limiting access to friends	All behavior put in place to limit the possibility of meeting friends during the relationship
Oppression	The feeling of being oppressed by judgments or behavior of the partner
Verbal abuse	Blatantly offensive language designed to humiliate and gain power over another person
Yelling	Behavior such as screaming
Threats	Intimidation’s acts to instill fear and insure compliance
Slandering	False spoken statements about someone that damages their reputation
Humiliating	Occasions or situations in which participants feel mortified and ashamed.

Below we present some selected quotations to illustrate the main categories emerging from narrative analyses. We chose both men and women’s citations to better underline the conformity in meaning.

Both men and women complain of limiting access to friends, often associated with irritability in the partner:

I could not meet females ... my friend lost her husband and I could not even invite her home, because all my female friends were sluts but all her male friends were perfect (M., man)
I have to be careful if I talk to someone, a friend, he understands badly, that is ... he gets nervous (G., woman)

A second element that both reported is the sense of oppression from ex-partner and their family:

I always felt high level of suffocation (P., man)
I did not feel free to make choices because his parents, his father and his mother, they were very pressing ... that is, they gave advice that then they turned into obligations (A., woman)

Verbal abuse includes both insults, both oral violence that affects the ex-partner in her fragility:

He said me that I was a bitch, with statements such as … woman of shit, with statements such as bitch ... insults, on insults, on insults, all in front of the child (P., woman)
She wrote me some messages “I have a family and you have not” (E., man)

Another common category is yelling:

Her phone calls, her screams and these things make me sick, she was yelling and this hurt me (R., man)
For a stupid thing he raised his voice very strongly, he yelled (L., woman)

Men and women report to be under threat:

He intimidated me, he intimidated also my children, he sent photos (V., woman)
*She said to me: “Look … your dog has finished eating, or you give me money, or you buy him food or I allow him to starve to death, do you know that?”* (M., man)

Slandering comprises false and defamatory statements perceived by partners:

I meet some friends, and they say that he goes around saying that I’m a whore, in front of my daughter (V., woman)
It is been more than ten years since she accuses me that … I’m alcoholic, she called all my friends to say this (R., man)

The last category of domestic violence presented in both narratives of men and women is Humiliating:

I woke up one morning that there was this girl to sleep at home, I was in the children’s room, when I woke up, he (the ex-partner) was in bed with her, he was comforting her ... but you cannot go to console another woman at home! With your wife! In underwear to console another woman ... humiliating! (I., woman)
She continued to argue that she was not my daughter, but I am the father, so ehm … I insisted “Do the DNA test and then you’ll find out what will happen!”. Why do you humiliate me so much? (L., man)

**FIGURE 1:**
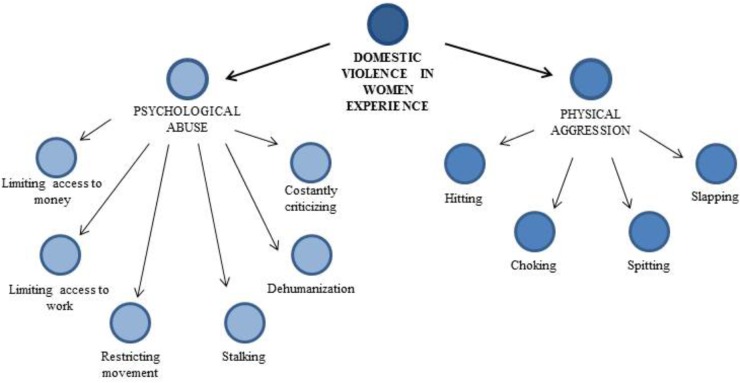
Domestic violence experienced only by women.

While men and women may experience common domestic violence behaviors, there are also some important differences. Below we present the results related to the second aspect highlighted by the analysis: the domestic violence experienced exclusively by women. **Figure 1** shows a graphical representation that summarize the Domestic Violence categories present only in women experience; it comprises both psychological and physical abuse.

Psychological abuse narrated by women comprises different categories: limiting access to money, limiting access to work, restricting movement, stalking, dehumanization, constantly criticizing. We describe these issues by quoting some sentences from women transcriptions.

Women narrated violence related to limiting access to money, that created a condition of dependence, as described in the words of this woman:

*I was economically dependent on him in the sense that I was not free to do shopping and ... I could not buy things without previous authorization*. (L., woman)

Behind this dependence is hiding the request to do something to access the economic resources:

*I could not buy even a underwear, all things that I want to buy should be inside an exchange agreement ... If I did housework, if I did something for him and his family, then it was possible that he approved my shopping or that he decided to buy me something*. (G., woman)

This limitation contributes to women perception to be not equipped to face the social reality:

*He had the management of the woman, that is ... I do not have a contact with real world, I do not know what is a bill; I do not know anything about these things, he has always done everything.* (G., woman)

The Limitation of access to work is a strong reason for hostility:

*We began to have fights for the money, because I started working and he told me that he brought me here (from my country) and that I had to give him money back*. (E., woman)

Women describe the Restriction of the movements as a psychologically violent act of control:

*He managed to confiscate even my house keys and my phone because I cannot leave the house”*. (D., woman)

In the women narratives emerge the story of some episodes of Stalking:

*He followed me, controlled what I did, not only controlled me: check at all the people who stood next to me, that is really the impossible*. (P., woman)
*When he called me twelve times to day, fifteen times a day, not answering the phone meant that he invaded everywhere*. (D., woman)

The narratives of women revealed also Dehumanization, in particular objectivation and animalization:

*At home I was just used, like ... not like a human being*. (L., woman)
It made me feel like a non-person; I didn’t have more my personality, I was a zombie. (M., woman)
*He took the keys of the house, he locked the door and he told me: “now you stay there for a while!”... honestly I felt like ... a package.* (P., woman)
*He treated me worse than a dog.* (N., woman)

Finally, in women’s verbalizations, the Constant criticism is perceived as a violence that threats personal identity and beliefs. The criticisms refer to different aspects of the person, as reported by this woman:

*He said me that I was disgusting, I dressed badly, and … in his opinion I did not behave well in everything, from silly things to important ones.* (R., woman)

These critiques seem to undermine these women, as it clearly emerges from the following sentence:

*He was constantly telling me that I did not know how to do things, he told me that I was not pretty enough, that I was not good enough and then … there was a moment that … this caused me distress.* (T., woman)

Some women point out that the criticism appeared associated with behaviors of little relevance:

*He, for a trifle, for a light on, for an overturned sugar, for a stupid thing he raised his voice very loudly, he did not say bad words, but had some little phrases that hurt me: “you’re brainless!”*. (L., woman)

This constant judgment does not seem to end with the separation, but rather increases:

*“he was always judging me, has always judged me, he has always blamed me, and still now he continues to make it more and more than previously”.* (C., woman)

The presence of physical violence emerges exclusively in the narratives of women. This kind of violence is articulated in different forms: hitting (“*he* *puts his hands on me three times*”), choking (“*he* *puts his hands on my neck*”), spitting (*“he spits in my face, I consider it an extreme cowardice*”). Physical violence is often narrated in association with the verbal violence as evidenced by this phrase:

*He kicked me and he started insulting me* (R., woman).

Physical violence generates a sense of fear in the women who have suffered it:

*He is a violent person who has put his hands on me so many times and, therefore, he frightened me* (S., woman).
*Fear is so much ... fear of even yes to be …to suffer certain things again ...* (D., woman)

**FIGURE 2:**
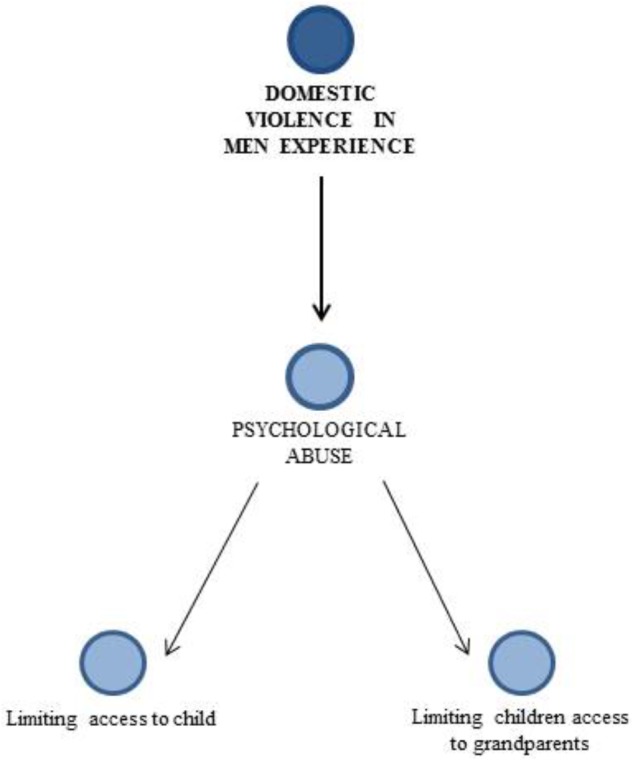
Domestic violence experienced only by men.

Domestic violence present only in men experience is represented in **Figure 2**.

For men, the main violence is related to limiting access to child. They attribute this to a sudden decision:

*Suddenly she decided that I couldn’t see the baby*. (G. man)

In the verbalizations of men emerge the idea that the ex-wives are not only involved in limiting contact in everyday life:

*The child saw me and he attempted to greet me; but she positioned herself in the middle.* (Y., man)
Today he told me that she is going to take the child from school when I was already in agreement with the child that I would take him and then we would go to play football. She is uncooperative in any event (L., man)

But, in men’s narratives emerge the idea that ex-wives aim precisely to eliminate father figure in their son’s life:

*She also wanted that I renounce to my parental authority, I absolutely could not ... because M. (the daughter) is ... she’s a part of myself and therefore, I could never and ever give up on M.* (G., man)

Another father says:

*She is doing everything to distance me from the child ... I asked her to see I. (the daughter) … I think this is my father’s right.* (F., man)

Men perceive this behavior as revenge:

*Depriving a daughter of the relationship with her father … I think that this is her revenge toward me*. (A., man)

In addition to the control exercised on the relationship between self and children, men report a limitation imposed also on the meeting with the grandparents and then access to the paternal lineage:

*She constrains me not to take the child to the grandparents, I cannot understand it! As her mother and father are G. (the son) grandparents the same is for mine! The child has the right to see her parents same as to see mine!* (E., man)

## Discussion

This work provides an original contribution to the field in order to understand the complexity and the characteristics of domestic violence associated with the separation context and to explore the specific gender differences regarding this topic.

The findings suggest that there is a common area of domestic violence perceived by both men and women and that concerns psychological aggression. In this area, consistent with World Health Organization [WHO] (2002) and Longobardi (2017) there are present some categories related to the use of violent verbal communication with the intent to harm or to exert control over another person. Men and women emphasize different forms of verbal assault and the use of intimate knowledge for degradation. This form of destructive communication could be considered specific of separation context in which violence occurs with continuity. Previously search identified emotional abuse as the most shared form of IPV (Karakurt and Silver, 2013), this area appears to be present both in men’s and women’s narratives also in the context of separation. They narrate to be subjected to threats, and exposed harm inflicted on victim’s pets, it can be emotionally abusive, causing distress to both humans and animals (Faver and Strand, 2007).

The results of this work also show that there are two main gender differences to consider.

The main difference that emerges from the analysis of the transcriptions of men and women is related to the presence of physical violence suffered exclusively in the narratives of women. Research on IPV in women has mainly paid attention to their victimization, for very valid reasons (Straus, 2006; Houry et al., 2008; Holtzworth-Munroe et al., 2010). In line with previous researchers women have often been considered to be the predominant victims and men the perpetrators of IPV. This could be coherent with traditional Italian family pattern in which women are devoted to child and housework and are considered weaker than men. Past findings highlight that severity, motives, and impact of IPV may be due to a gender asymmetry. Men often initiate and perpetrate more severe IPV which leads to more serious consequences or injuries (Ko Ling, 2011).

In the narratives of men no episodes of experienced physical violence appear. This confirms the literature trend that focuses on male-to-female violence, while overlooking female-to-male violence.

Specifically, regarding psychological violence experienced by men and women our results highlight a more complex scenario. Women identify a wide variety of types of domestic violence suffered, while men recognize only a few behaviors. A possible explanation is about social desirability. Men have to maintain their position in society. Face-to-face reporting of IPV behavior may induce shame, guilt, and embarrassment, which possibly lowers the likelihood of disclosure of such violence (Felson and Paré, 2005). So men may have trouble reporting certain behavior.

Women complain about the violence that affects control (money, work, movement) and aspects that undermine the identity (dehumanization and criticism).

In the context of separation more than in other condition controlling a battered person’s access to work and financial resources can directly affect their possibility to separate. Men should implement also other form of violence referred by women, as a tactic to insure compliance. Minimization, denial, and blame destabilize the credibility and identity of battered/abused individuals. This appears particularly significant in relationship breakdown because people could loose their certainties.

Gender difference in this kind of context develops a reflection on the fact that also women are perpetrators of violence even if in the literature this perspective is less treated.

In our study, men reported a specific domestic violence perpetrate from women: the limiting access to meet children. This violence includes threats and/or behavior of exclusion from whole father ancestry. The narratives in this topic recall the concept of Parental Alienation (Gardner, 2002). Psychological studies focused on this specific syndrome considering it a form of psychological child abuse, that can lead to long-term traumatic psychological and physical effects in the children (Cavanna, 2013; von Boch-Galhau, 2018). Only recently a particular attention has been given to parent perspective (Balmer et al., 2018), underlying an intense psychological distress as a result of being alienated from their children. Data of our work suggest that specifically women participants use this type of violence, and that men experience significant exposure to parental alienation tactics. This finding is consistent with previous research (Bow et al., 2009).

A final reflection on the specificity of violence highlights that men use violence that affects the relationship with the outside (money, work), the possibility of autonomy (movements) and the definition of identity. On the contrary, women perpetrate violence in the area of relationships with their children. This seems in line with gender stereotypes, resurrecting or reinforcing the division between male-dominated public spaces, and the private spaces defined as women’s domain (Scabini and Cigoli, 2006). Man threatens woman in the aspects on which she is weaker (e.g., women earn less, are less independent) and woman exercises a deprivation where man is more fragile. On the basis of gender differences in affect, behavior, and cognition (self-construal, emotional experience, selective memory), according to Gardner and Gabriel (2004) women would pay attention more on the relational aspects of interdependence in close relationship; this aspect could increase the women vulnerability to the effects of domestic violence.

### Limitations and Strengths of the Study

There were, of course, some limitations to this study, first the study does not consider the dyadic dimension as interpretative paradigm of the relationship between violence and gender as suggested by the Johnson and Ferraro (2000) studies. This should be useful to better understand the relational context surrounding IPV.

Second, data analysis in qualitative research is inherently subjective. We collect data in a Family Mediation Center, therefore, we may have missed the more severe levels of IPV.

Furthermore, the results are based on narratives consequently recall bias or unwillingness to report may influence the findings. Within the context of these limitations, however, our study suggests some possible practical implications for operators and procedures regarding this type of context to enhance programs that can empower women and men.

The strength of the present research was to analyze IPV in the context of separation, highlighting common and specific area. Within the narratives of the Italian couples, the common categories were emphasized and gender differences underlined. The narratives of the participants made it possible to highlight that the main differences are about the perception of physical violence only in women words.

As the separation can be considered a risk factor for intimate violence, our findings could be very useful because only a few studies have investigated the domestic violence in separated couples. Additionally, this study supports the need for further and more in-depth research on the gender differences in how IPV is used by men and women in different transition of family life cycle.

A greater understanding of similarities and differences in the conceptualization of domestic violence by gender can help to improve appropriate gender specific interventions and prevention efforts.

## Conclusion

Our findings show some types of experienced IPV characteristic of separated couples in Italian context and underline some gender specificity in men’s and women’s narratives about this topic. This first exploratory study raises many questions that are not sufficiently studied, but that need to be addressed about the different form of IPV carried out or suffered from men and women in the context of relationship breakdown. If men and women differ in their conceptualizations of IPV, this suggests that interventions could be begin with the creation of a greater awareness and a common understanding about the problem, especially for non-physical violence. This common understanding is even more important and meaningful in the delicate phase of separation in which sharing is made difficult by the transition itself.

Jaffe et al. (2008) recommend that differing types and levels of IPV should be incorporated into case analyses and choices regarding mediation. The mediator must pay attention to the imbalance of power that could be generated between partners and he must also have sufficient power to intervene on couple dynamics, identifying forms of IPV. The necessary first step to ensuring the safety of mediating parties must therefore be detecting a history of IPV, and the present study suggests that we have to take into account of specificity gender matter (Ballard et al., 2011). An interesting result concerns a very particular form of violence that women exercise in case of separation and that regards the limitation of father’s meetings with children that often causes parental alienation syndrome. This represents a central issue in mediation practice.

This article should contribute to the growth of the literature in Italy and provide interesting suggestion for other international context that are facing the domestic violence phenomenon. As well as data on domestic violence in the Italian context are collected exclusively on women, also in international studies the dominant portrayal of domestic violence does not cover men as victims, with rare exceptions (Costa, 2017).

This work suggests possible practical implications for researchers, clinicians, and procedures regarding this type of domestic violence, to enhance intervention programs. In fact, these findings could indicate two possible reflections and work areas: first, also men must be considered victims, and clinicians are called to promote communication and emotional expression about violence. Second, operators should promote empowerment paths for women to strengthen their identity, self-esteem, and self-efficacy as protection of the self from the abuses of the other.

## Ethics Statement

This research was conducted following the ethical norms stipulated by the Italian Psychology Association (AIP). Before the narrative task, written informed consent was obtained from all participants in accordance with the Declaration of Helsinki. It contained a brief explanation about the research and informed potential participants that the interview would be audio-recorded and the data processed and anonymized; it also assigned a code to each participant, in compliance with Italian Law on Privacy no. 196/2003. Research ethics committee has not yet established in the authors’ institution when the research started, so an ethics approval was not required for this research as per the authors’ Institutions’ guidelines and national regulations.

## Author Contributions

DC and FG conceived of the presented idea and supervised the findings of this work. PC developed the theory, performed the qualitative analyses, and wrote the manuscript with support from LM and FB. All authors discussed the results and contributed to the final manuscript.

## Conflict of Interest Statement

The authors declare that the research was conducted in the absence of any commercial or financial relationships that could be construed as a potential conflict of interest.
